# Current Perspective on the Role of the Circadian Clock and Extracellular Matrix in Chronic Lung Diseases

**DOI:** 10.3390/ijerph20032455

**Published:** 2023-01-30

**Authors:** Kameron Hahn, Isaac Kirubakaran Sundar

**Affiliations:** 1 Department of Biological Sciences, University of Missouri, Columbia, MO 65211, USA; 2 Department of Internal Medicine, Division of Pulmonary, Critical Care and Sleep Medicine, University of Kansas Medical Center, Kansas City, KS 66160, USA

**Keywords:** circadian clock, lung disease, extracellular matrix, fibrosis, TGFβ signaling

## Abstract

The circadian clock is a biochemical oscillator that rhythmically regulates physiological and behavioral processes such as inflammation, immunity, and metabolism in mammals. Circadian clock disruption is a key driver for chronic inflammatory as well as fibrotic lung diseases. While the mechanism of circadian clock regulation in the lung has been minimally explored, some evidence suggests that the transforming growth factor β (TGFβ) signaling pathway and subsequent extracellular matrix (ECM) accumulation in the lung may be controlled via a clock-dependent mechanism. Recent advancements in this area led us to believe that pharmacologically targeting the circadian clock molecules may be a novel therapeutic approach for treating chronic inflammatory lung diseases such as asthma, chronic obstructive pulmonary disease (COPD), and idiopathic pulmonary fibrosis (IPF). Here, we update the current perspective on the circadian clock role in TGFβ1 signaling and extracellular matrix production during chronic lung diseases.

## 1. Introduction

The mammalian circadian clock is a 24 h cycle of physiological and behavioral processes. Daily fluctuations of external stimuli (i.e., light/dark cycles) working together with a molecular circadian system regulate many organ systems and play a prominent role in sleep–wake cycles, metabolism, body temperature, immune response, and inflammation [1,2,3,4]. Accordingly, disruption of the circadian clock has been linked to many chronic diseases of the liver, heart, kidney, brain, and lungs [5,6,7,8]. Ongoing research work in our laboratory is specifically focused on addressing how growth factors and inflammatory signaling pathways within the lung are rhythmically regulated by the circadian clock. It is well known that symptoms of chronic lung diseases including asthma and COPD worsen depending on the time of day, which has sparked interest in understanding how the circadian clock regulates other chronic lung complications including cystic fibrosis (CF), pulmonary arterial hypertension (PAH), and idiopathic pulmonary fibrosis (IPF) [9].

Remodeling of the lung extracellular matrix (ECM) is a prominent phenotype in the pathobiology of chronic lung diseases including asthma, COPD, and IPF [10]. The ECM in the lung is a network consisting of collagen, enzymes, and glycoproteins that surround cells of all solid tissue including those of the lung. Under homeostatic conditions, the ECM is very important for providing structural support and communication pathways between cells, but poor ECM remodeling can have drastic consequences for lung health [11]. In IPF, exacerbated epithelial-to-mesenchymal transition (EMT) and fibroblast-to-myofibroblast transition (FMT) are associated with an increased inflammatory response and excessive ECM deposition, which cause thickening and scarring of the lung tissue. Myofibroblasts are an activated form of fibroblast that produce excessive amounts of inflammatory cytokines, chemokines, and growth factors which aid in collagen deposition and ECM remodeling [12]. One key growth factor released by these activated fibroblasts is transforming growth factor-β1 (TGFβ1), which plays a major role in ECM remodeling and the subsequent pathogenesis of chronic lung diseases [13]. Interestingly, one study has shown that the induction of TGFβ1 augments the expression of *Bmal1*, which is required for TGFβ1 signaling in the fibrotic lung [14]. The interplay between clock genes and mediators of chronic lung disease is poorly understood, but some research indicates that this relationship could be targeted for the treatment of chronic lung disease. A recent study suggested that targeting the circadian clock component REV-ERBα with small-molecule drugs could be a viable therapeutic approach for pulmonary fibrosis [15]. This review will focus on the circadian regulation of TGFβ1 signaling and ECM remodeling in the lung and how we can target the circadian clock as a novel treatment option for chronic inflammatory lung diseases.

## 2. The Circadian Clock Role in Chronic Lung Disease

The mammalian circadian clock is a biological timer that coordinates many physiological and behavioral processes. This clock can be divided into two major systems: central and peripheral. The central clock is the command center located in the suprachiasmatic nucleus (SCN) region of the hypothalamus in the brain. The SCN is also known as the “master clock” because of its regulatory role in clock oscillations. The SCN registers an input of information such as light, processes this information, and relays it to other parts of the brain as well as the peripheral organs in the form of hormonal and neuronal signals. The most well-known response to light/dark signals is our daily sleep schedule, but the circadian clock is much more diverse than sleep alone. The circadian clock controls most, if not all, peripheral organ systems in the body. The key organ systems that have shown dependence on circadian cues are the liver, heart, kidney, brain, and lung [5,6,7,8]. Within these peripheral organ systems, the circadian clock plays a prominent regulatory role in controlling several vital biological processes such as metabolism, body temperature, immune response, and inflammation [1,2,3,4]. Undoubtedly, disruption of the circadian clock can lead to many chronic diseases and disorders.

The regulation of circadian physiological responses begins at the molecular level. Within nearly every cell throughout the body, there exists a molecular clock: a transcriptional/translational negative feedback loop of clock genes. In this feedback loop, a heterodimer is formed between transcription factors CLOCK:BMAL1 which increases the expression of period proteins (PER1/2/3) and cryptochromes (CRY1/2) by binding to the E-box (transcription enhancer element). PER and CRY proteins dimerize and translocate to the nucleus where they interact with CLOCK:BMAL1, resulting in the suppression of their own transcription. PER and CRY proteins are then degraded via polyubiquitination. Consequentially, lower levels of CRY and PER proteins restore CLOCK:BMAL1 transcriptional activity, and the cycle repeats [16]. In a secondary feedback loop, the nuclear receptors REV-ERB and ROR (retinoid-related orphan receptor) stabilize clock timing by repressing and activating *Bmal1* expression, respectively [17,18].

Accumulating evidence suggests that the circadian clock plays a major role in lung function. Symptoms of asthma and COPD have long been associated with a daily cycle of activity, and recent studies suggest that molecular clock disruption exacerbates the pathogenesis of other chronic lung diseases such as IPF, CF, PAH, and lung cancer [9,19,20]. Not only has circadian clock disruption been linked to the development of chronic lung disease, but researchers have even explored the idea of targeting the circadian molecular clock to combat inflammation and lung remodeling involved in asthma and IPF [15,21]. The mechanism behind the molecular clock regulation of inflammatory and fibrotic response is poorly understood, but some evidence may suggest that ECM remodeling is involved.

## 3. The Multifactorial Role of Extracellular Matrix in Chronic Lung Disease

The extracellular matrix is a network surrounding the cells that functions to maintain cell structure, provide communication pathways between cells, and influence cell behaviors such as migration, proliferation, and apoptosis. In most tissues, the ECM is made up of three components: proteoglycans, fibrous proteins, and integrins. Proteoglycans are a web of polysaccharides and proteins that hold other ECM components in place. Fibrous proteins form connections between cells and including the glycoproteins collagen, fibronectin, laminin, and elastin, which come in various forms. These proteins determine the elasticity, hardness, and strength of the tissue. Integrins are receptors found on the cell that connect ECM activity with cellular responses by relaying information to microfilaments within the cell which can even alter gene expression [22]. ECM components are secreted by specialized cells, making their composition very tissue specific. For example, bone ECM is deposited by osteoblasts which favor a harder, dense matrix surrounding the cells. Alternatively, normal lung fibroblast ECM deposition results in a softer, more elastic ECM structure.

ECM remodeling in the lung is necessary for maintaining the proper tissue structure and morphogenesis, but aberrant remodeling can contribute to the development of chronic inflammatory lung diseases. In severe asthma, airway obstruction is associated with ECM deposition in the submucosal regions of the lung [23]. Even in the absence of inflammation, bronchoconstriction has been demonstrated to result in airway remodeling, providing evidence for an ECM-dependent remodeling response in asthma [24]. Recent studies suggest that lysyl oxidase-like-2 (LOXL2), a matrix crosslinking enzyme, as well as integrin-mediated cell–cell and cell–matrix interactions, play a vital role in matrix stiffening involved in airway remodeling [25,26]. In lung fibrosis, EMT and FMT are processes that lead to increased ECM deposition. Myofibroblasts are an activated form of fibroblast highly prevalent in pulmonary fibrosis that express high levels of alpha-smooth muscle actin (αSMA), and secrete large amounts of collagen, fibronectin, and other ECM proteins [27]. Understanding the role of the ECM in regulating cell structure, behavior, and communication is crucial for understanding the pathogenesis of these chronic inflammatory lung diseases.

One characteristic of the ECM concerning chronic lung diseases that has been minimally discussed is the communication pathway it provides between cells. Some recent studies have explored exosome activation and extracellular vesicle (EV) transportation of ECM components in COPD, bronchopulmonary dysplasia (BPD), and lung cancer progression [28,29]. EV communication pathways seem to facilitate ECM deposition, but alternatively, one study showed that mesenchymal stem cell exosomes inhibited ECM production in keloid fibroblasts [30]. The interesting role of EVs/exosomes in ECM remodeling should be further studied to understand how they propagate chronic lung diseases or even how they could be repurposed to combat the progression of lung diseases such as severe asthma, COPD, and IPF.

## 4. Transforming Growth Factor-β Signaling in ECM Remodeling and Chronic Lung Disease

Transforming growth factor-β1 (TGFβ1) is a multifunctional cytokine that is a well-known mediator of FMT and a major player in the development and progression of pulmonary fibrosis [13]. TGFβ is secreted by many cell types including macrophages, lymphocytes, platelets, osteoblasts, and fibroblasts [31,32,33]. In fibrosis, fibroblasts secrete a latent form of TGFβ1 that is stored in the extracellular space until it is activated by integrins, proteases, and other mechanisms [34]. After activation, TGFβ1 binds to the membrane-bound receptor complex TGFβR-I/II, initiating the phosphorylation of SMAD2/3, the inhibition of glycogen synthase kinase 3 beta (GSK3β), and β-catenin translocation to the nucleus, where cell-specific gene alterations occur [13]. SMADs alone are known to play major roles in cell development and growth, and the SMAD2/3 complex has shown importance in the downstream TGFβ1 signaling that drives FMT [35]. GSK3β and β-catenin are key components of the Wnt/β-catenin signaling pathway, which is known to be involved in embryonic development and the homeostasis of adult tissues, and recently has been linked to lung fibrosis [36]. Wnt ligands are glycoproteins that initiate the signaling pathway by interacting with a frizzled membrane receptor [36]. Without active Wnt signaling, β-catenin is degraded by GSK3β, which hinders the signaling process, but active Wnt signaling phosphorylates and degrades GSK3β, which results in the accumulation of β-catenin within the cell and successful signaling [36]. Interestingly, blocking Wnt/β-catenin signaling has been shown to decrease TGFβ1-induced myofibroblast and ECM production [37], and the Wnt/β-catenin pathway has shown evidence of converging with downstream TGFβ1 signaling resulting in increased fibrotic phenotypes [38]. Furthermore, a prior study revealed that TGFβ1 gene expression is regulated by the mechanical tension of protein fibers that compose the ECM [39]. With this knowledge, Wnt/β-catenin and TGFβ1 signaling crosstalk and mechanical ECM influence may be important in the development of fibrotic lung disease. A more recent study explored a positive feedback loop beginning with TGFβ1 and SMAD3 signaling followed by increased gene expression of the sushi-repeat-containing protein, X-linked 2 (SRPX2), resulting in increased SMAD3 phosphorylation and subsequent TGFβ1-mediated profibrotic response [40]. This and similar TGFβ1-mediated signaling pathways alter gene expression within lung fibroblasts, which leads to αSMA-induced tension, along with the deposition of collagen and other ECM factors. Understanding the TGFβ1 signaling mechanism is important in lung fibrosis, which begins with ECM tension and subsequently extensive ECM deposition.

## 5. Circadian Clock Modulation of the ECM

### 5.1. Circadian Modulation of the ECM throughout the Body

There is sufficient research to suggest that the ECM plays important roles in tissue structure, communication pathways, and cell behavior. Accordingly, the ECM is often a factor in dysfunctional cellular and tissue processes. While the importance of the ECM is known, there is a gap in knowledge on the true controller of the ECM. A potential ECM modulator is the circadian clock. Regulation of the ECM components by circadian clock genes and related processes have been demonstrated in various tissues including the nervous, squamous epithelium, connective/cartilage, liver, kidney, and cardiac muscle [41,42,43,44,45,46,47,48,49,50]. Circadian regulation of the ECM has been minimally studied in the lung, but analyzing similar processes and structures in different tissues could be a starting point for future investigations. Multiple studies suggest an important role of circadian genes including *Cry2* in connective tissue homeostasis, chondrocyte function, and cartilage formation [44,45,46]. Another study showed that deletion of the clock gene *Per2* may increase liver fibrosis in mice [47]. Deletion of *Bmal1* increased inflammation, ECM deposition, and exacerbated fibrosis in mice cardiomyocytes [50]. Furthermore, recent findings suggest that EVs and the protein cargo they carry may be circadian-regulated [51,52]. In the squamous epithelium, recent evidence suggests that microRNAs function as intercellular communicators for wound repair processes through transportation via extracellular vesicles [43]. Although various tissues throughout the body differ functionally, many molecular and intercellular processes are maintained universally. Reviewing studies in different tissues has shown circadian regulation of ECM components related to wound repair, fibrosis, inflammation, and cell–cell communication via EVs. All such processes are drivers for chronic inflammatory lung disease and provide an interesting area for potential research.

### 5.2. Circadian Modulation of ECM in Chronic Lung Disease

The circadian clock in chronic lung disease has been minimally studied, but evidence suggests that the ECM may be one of the key players in the dysfunction of lung tissue. We have previously explored the nuclear heme receptor and molecular clock transcription inhibitor, REV-ERBα, in cigarette smoke (CS)-induced mice models. These studies concluded that there are lower levels of REV-ERBα protein in smokers and COPD patients and REV-ERBα inhibition worsens CS-induced inflammation [53,54]. While these studies demonstrated an important role of REV-ERBα in lung inflammation and related diseases, the mechanism behind it remained unexplored. A more recent study provides additional evidence that REV-ERBα regulates CS-induced pulmonary inflammation as well as EMT *in vivo* in mouse lungs [55]. They showed upregulated transcript levels of EMT markers (vimentin, TGFβ1, SERPINE1, collagen type III α 1 (Col3a1), matrix metalloproteinase 2 (MMP2), and TIMP metallopeptidase inhibitor 1 (TIMP1)) following CS exposure and they are directly associated with the ECM [55]. Additionally, there was a significant increase in airway collagen deposition in CS-exposed REV-ERBα knockout (KO) mice when compared to CS-exposed wild-type (WT) mice, suggesting that REV-ERBα may inhibit collagen production [55].

Some recent studies also support the ECM–circadian clock axis. One of these studies looked at the preventative role of tetraspanin Cd151 in pulmonary fibrosis and emphysema. Cd151 is a type of membrane scaffolding protein that may modulate α3β1 integrin-dependent adhesion and migration and promote endothelial adhesion [56]. This study demonstrated that Cd151 KO was associated with downregulated circadian clock genes (*Cry1*, *Per2*, and *Per3*), and upregulation of lysyl oxidase (*LOX*), collagen type III alpha I (*COL3A1*), microfibrillar associated protein 5 (*MFAP5*), and elastin (*ELN*) which are all ECM components [57]. The mechanism of communication between tetraspanin Cd151 and ECM components may be subject to circadian clock regulation, which is another interesting yet unexplored area of study.

More recent efforts have gone into exploring cellular mechanisms of ECM accumulation in pulmonary fibrosis. One study concluded that the peroxisome proliferator-activated receptor alpha (PPARα) agonist pemafibrate attenuates pulmonary fibrosis by inhibiting myofibroblast differentiation and ECM accumulation [58]. PPARα is a nuclear receptor dependent on ligand activation that enhances the transcription of anti-inflammatory cytokines and inhibits the production of the proinflammatory cytokines TNFα, IL-1β, and IL-6 [59]. Protein analysis showed that bleomycin-challenged mice treated with pemafibrate had reduced ECM components collagen-1 and fibronectin [58]. Interestingly, a previous study demonstrated that PPARα is expressed in a circadian manner and regulated by the core circadian protein CLOCK [60]. These findings together provide a better understanding of PPARα and its role in lung fibrosis, demonstrating how there could be a crosstalk between the circadian clock and ECM production.

To further explore the circadian clock regulation of ECM production in chronic lung disease, we have analyzed the ECM and ECM-associated genes that may be regulated by the circadian clock (Table 1). Here, we utilized two different databases (Matrisone DB (http://www.pepchem.org/matrisomedb, accessed on 25 January 2023) and CircaDB (http://circadb.hogeneschlab.org/, accessed on 25 January 2023)) which were cross-referenced to evaluate the rhythmic expression of ECM genes that may be associated with lung fibrosis. MatrisomeDB is a database that has gathered experimental proteomic data to examine the distribution of ECM proteins in various tissues, including significant data from studies on fibrotic lung tissue in mice [61]. These results were filtered and exported to gather a list of ECM and ECM-associated genes that may be highly expressed in the lung. The distribution of ECM protein expression in the fibrotic lung tissue of mice is based on a calculated confidence score of peptide-to-spectrum matches (PSMs) [61]. A higher confidence score is evidence of a more prevalent expression of the protein in the target tissue. It is important to note that Table 1 is not an exhaustive list of ECM-related proteins, but rather a simplified representation of the highest confidence scores presented by MatrisomeDB. TGFβ1, for example, was one of the proteins demonstrating high expression in fibrotic lung tissue of mice, but the confidence score was too low to meet our parameters for inclusion in Table 1.

**Figure 1:**
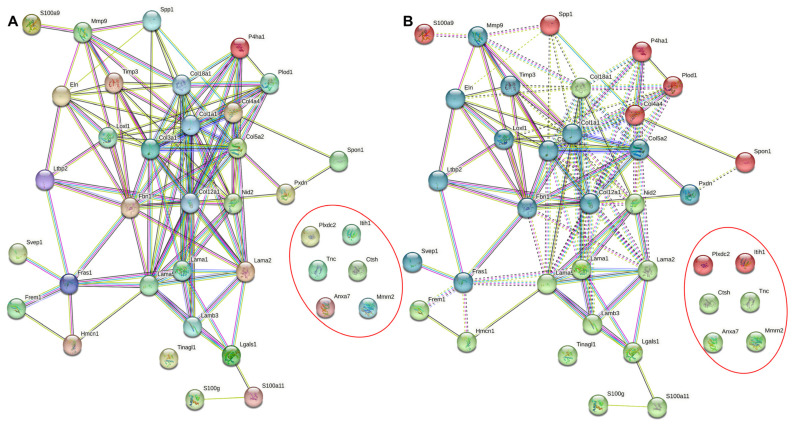
**STRING database network analysis of rhythmic mouse ECM and ECM-associated protein targets.** (**A**) STRING DB analysis without clustering. (**B**) STRING DB analysis with K-means clustering revealed network contains 37 nodes, 116 edges, an average local clustering coefficient of 0.506, and a PPI enrichment *p*-value < 1.0e-16. The confidence score threshold was set as 0.4 (medium) for the analysis. Coloring in Figure 2A holds no significant meaning while coloring in Figure 2B (red, green, blue) represent clusters of proteins that are similar based on a machine-learning algorithm. The circled proteins (not connected) are similar enough to be clustered in the network but have not shown any evidence of association. The lines or edges between protein nodes represent evidence of association characterized by color: fusion (red), neighborhood (green), co-occurrence (blue), experimental (purple), text mining (yellow), database (light blue), and co-expression (black). Functional enrichment in the network specific to the top 5 biological processes, molecular functions, and cellular components including KEGG pathways are summarized in Table 2.

Another database, CircaDB, was used to determine if any of the ECM-related protein coding genes that are highly expressed in the lung show substantiation of circadian transcription rhythms. CircaDB has gathered mouse studies using Affymetrix microarray analysis over a long period to measure rhythmic gene expression [62]. By cross-referencing MatrisomeDB and CircaDB, we were able to tabulate the highly expressed ECM-related protein genes in fibrotic lung tissue of mice that showed rhythmic expression in the lungs (Table 1). To analyze the interactions of ECM proteins in the lung, the STRING database was used to organize proteins by their physical and functional associations. STRING DB networks of the rhythmically expressed ECM proteins established from Table 1 consisting of analysis with and without clustering containing 37 nodes and 116 edges are presented in Figure 1A,B. Based on this information, we can see that collagen, fibronectin, and laminins (ECM proteins) demonstrate high levels of physical and functional association. The functional enrichment in the network specific to the top five biological processes, molecular functions, and cellular components including KEGG pathways is summarized in Table 2. To no surprise, these processes, functions, and components are strongly related to the ECM and related structural properties. This information may be useful to explore how the circadian clock regulates the functional proteins that make up the ECM and how specific proteins could be targeted through circadian mechanisms to produce a physiological response within the ECM.

## 6. The Circadian Clock Influence on TGFβ Signaling in Chronic Lung Disease

TGFβ is a key player in lung fibrogenesis, so this is a good starting point for understanding the lung circadian clock–ECM axis. Another study explored the interplay between the circadian clock and TGFβ1 mechanisms. They conducted a qRT-PCR analysis of lung tissues from TGFβ1 adenovirus-treated mice which showed an upregulation of *Bmal1* and *Npas2* and downregulation of *Per1*, *Per2*, *Per3*, *Rev-erbα*, *Rorα*, and *Dbp* [14]. In prior studies, BMAL1 was shown to be a strong regulator of TGFβ1 pathway components, so this study focused its efforts on this clock gene specifically. BMAL1 was shown to attenuate FMT and EMT, evidenced by the decreased αSMA, plasminogen activator inhibitor-1 (PAI-1), and E-cadherin (ECAD) gene expression and increased fibronectin containing extra domain A (FN-EDA) gene expression accompanied by attenuated MMP9 production [14]. Mechanistically, they found that BMAL1 regulates the degradation of GSK3β, which is an inhibitor of the TGFβ1/SMAD3 signaling pathway [14]. The regulation of this pathway by BMAL1 highlights the role of the circadian clock in TGFβ1-induced fibrogenesis and ECM production. However, not much is known about other core clock targets, such as the role of PER(s), CRY(s), and REV-ERB(s) in TGFβ-induced profibrotic phenotype and fibrogenesis in vitro and in vivo.

## 7. Targeting the ECM with Circadian Clock-Based Therapeutics for the Treatment of Chronic Lung Disease

**Figure 2:**
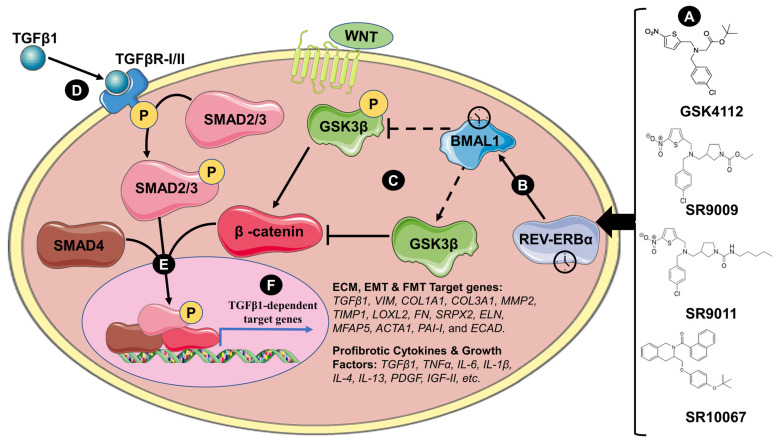
**Targeting the molecular clock using synthetic REV-ERB agonists to modulate TGFβ1-mediated ECM and ECM signaling mechanisms.** (**A**) Pharmacologically targeting the molecular clock using REV-ERBα agonists (e.g., GSK4112, SR9009, SR9011, SR10067); (**B**) REV-ERBα inhibits the transcription of BMAL1; (**C**) active WNT signaling phosphorylates GSK3β, which promotes its degradation. Phosphorylated GSK3β can no longer inhibit β-catenin activity. BMAL1 may function to inhibit phosphorylation of GSK3β to promote degradation of β-catenin; (**D**) active TGFβ1 binds to TGFβR-I/II to phosphorylate SMAD2/3; (**E**) phosphorylated SMAD2/3, SMAD4, and β-catenin form a complex that translocates into the nucleus; (**F**) the SMAD2/3, SMAD4, and β-catenin complex bind to the promoter region and enhance transcription of TGFβ1-dependent target genes including ECM components, EMT and FMT markers, and profibrotic cytokines and growth factors.

Emerging evidence supports that circadian clock-mediated regulation of ECM production and accumulation may be one of the possible mechanisms driving chronic inflammatory lung diseases. Some recent interest has been in targeting the circadian clock as a novel therapeutic strategy for chronic inflammatory lung diseases. Currently, we are exploring novel circadian clock-based therapeutics (small molecules that specifically regulate the transcriptional and translational activity of core clock targets) using translationally relevant *in vitro* and *in vivo* models for the treatment of chronic inflammatory lung disease. We have utilized small-molecule drugs SR9009 and GSK4112 (REV-ERB agonists) that target and activate REV-ERBα. The REV-ERBα agonist SR9009 reduced acute CS-induced lung inflammation and abnormal EMT of mice, and the other REV-ERB agonist, GSK4112, reduced TGFβ1-induced FMT in human fetal lung fibroblast 1 (HFL-1) [55]. Chronotherapeutic treatments, or time-based delivery of drugs, have been available and commonplace in the treatment of some diseases including bronchial asthma for some time now [63]. Our novel approach to circadian clock-based therapeutics of chronic inflammatory lung diseases aims to target and manipulate the core molecular clock using small-molecule drugs. Our current understanding of this mechanism is outlined in Figure 2, suggesting that the small molecules (GSK4112, SR9009, SR9011, and SR10067) may be administered to activate the clock protein REV-ERBα, subsequentially inhibiting BMAL1 transcription, blocking activation of Wnt/β-catenin and SMAD2/3 signaling, which may function to decrease FMT, EMT, inflammation, and ECM accumulation commonly seen in chronic lung diseases. While the REV-ERBα agonists we have presented are interesting and promising drug targets, there are other unexplored REV-ERBα agonists, antagonists, and a plethora of drugs that target other components of the molecular clock including RORs, CRY, SIRT1, GSK3β, and casein kinase enzymes [64]. Manipulating the circadian clock with target-specific small-molecule drugs is a novel approach to treating inflammatory diseases but is minimally researched regarding applications for chronic inflammatory lung diseases [65]. This new area of research provides a promising alternative to treating excessive inflammation, fibrosis, and ECM production exacerbated in asthma and pulmonary fibrosis.

The circadian clock plays a broad role in many body systems through both central and peripheral clock regulation, but our current understanding of lung-specific targeting is largely pragmatic. Before treating patients with novel clock drugs, we must address the current challenges including unforeseen off-target effects. A recent review highlighted the existing challenges and limitations in translating circadian medicine and chronotherapy and possible ways to overcome these challenges in the future [65]. Along with the ECM–clock relationship we have presented here, the circadian system has also demonstrated coupling activity with other important immune and metabolic pathways such as the hypothalamic–pituitary–adrenal axis; therefore, by altering the expression of circadian clock proteins, we should be cautious of disrupting these relationships and the threat of hypercortisolism [66]. For example, the small molecules (REV-ERB agonists: GSK4112, SR9009 and SR9011) were previously described to have promising effects for inhibiting inflammation and fibrosis of the lung [15,53,54,55], but the same drugs have been demonstrated to alter the metabolic regulation of obese mice and increase wakefulness, which could alter sleep schedules if not timed correctly [67,68]. REV-ERB agonists including SR9009 and SR9011 both modulate the molecular clock by reducing the amplitude of clock gene expression, which could have systemic responses if treatment is not localized [69]. Before moving forward with pharmacological treatment of the lung with clock drugs, we must first consider possible side effects and drug efficacy and actualize more site-specific targeting.

## 8. Conclusions

The mammalian circadian clock is a complicated system that continuously acts as the interface between our external environment and internal cellular processes. Any sort of dysregulation in the circadian timing system may have consequences for the body and vice versa, but with the finding of our recent literature review, we might be able to target and even regulate the function of the circadian system. When it comes to chronic lung disease, one of the major dysfunctional processes is ECM production. The extracellular matrix is a complex system that serves many functions, but abnormal and excessive ECM accumulation in the lung could lead to inflamed, fibrotic tissue evident in chronic lung diseases. Mounting evidence suggests that inflammation and fibrosis driven by ECM production may be regulated in a circadian manner through a TGFβ1 signaling pathway. It might prove beneficial to further study the circadian clock’s role in this pathway and explore circadian clock-based therapeutics to control ECM activity including excessive structural protein production and intercellular communication and transportation pathways.

## Figures and Tables

**Table 1:** **Rhythmic expression of mouse ECM and ECM-associated genes highly expressed in lung fibrosis**.

Gene Symbols	Gene Names	JTK Period	JTK Phase	JTK *p*-Value	JTK q-Value	Confidence Score
***Lama5***	Laminin, alpha 5	26.0	7.0	7.35e-06	0.0006	100,033
***Col1a1***	Collagen, type I, alpha 1	24.0	4.0	2.48e-10	3.53e-07	43094
***Lamb3***	Laminin, beta 3	22.0	12.0	0.001	0.020	35,555.7
***Col12a1***	Collagen, type XII, alpha 1	24.0	15.0	0.0001	0.006	25,065.2
***S100a11***	S100 calcium binding protein A11	22.0	9.0	0.001	0.024	24,752.1
***Eln***	Elastin	24.0	23.0	2.36e-07	5.35e-05	23,947.5
***Col3a1***	Collagen type III, alpha 1	24.0	2.0	3.78e-07	7.30e-05	20,549.2
***Lama2***	Laminin, alpha 2	24.0	7.0	3.24e-05	0.001	17,516.3
***Nid2***	Nidogen 2	24.0	16.0	2.36e-07	5.35e-05	13,971.3
***S100a9***	S100 calcium binding protein A9	25.0	2.5	0.0004	0.011	10,646.8
***Tnc***	Tenascin C	24.0	5.5	0.0002	0.007	7974.4
***Tinagl1***	Tubulointerstitial nephritis antigen-like 1	24.0	14.5	0.001	0.032	7707.24
***Hmcn1***	Hemicentin 1	26.0	6.0	0.0002	0.007	7150.28
***Itih1***	Inter-alpha trypsin inhibitor, heavy chain 1	24.0	4.0	0.002	0.040	5836.89
***Fbn1***	Fibrillin 1	24.0	2.0	8.88e-08	2.74e-05	5221.73
***Anxa7***	Annexin A7	24.0	9.0	0.0001	0.004	4549.21
***Pxdn***	Peroxidasin homolog	22.0	8.0	9.32e-07	0.0001	4398.3
***Col5a2***	Collagen, type V alpha 2	26.0	22.0	0.0003	0.009	3921.87
***Fras1***	Fraser syndrome 1 homolog	26.0	6.0	0.002	0.036	3695.19
***Col18a1***	Collagen, type XVIII, alpha 1	24.0	4.0	0.002	0.040	3506.24
***Frem1***	FRAS1-related extracellular matrix protein 1	26.0	8.0	0.0001	0.004	3063.51
***Mmrn2***	Multimerin 2	22.0	9.0	6.48e-05	0.003	2930.29
***Spon1***	Spondin 1	220.	19.5	0.002	0.036	2808.89
***Spp1***	Osteopontin	24.0	19.0	7.64e-07	0.0001	2684.34
***Ctsh***	Cathepsin H	24.0	17.0	0.0002	0.007	2621.88
***Lgals1***	Lectin galactose binding, soluble 1	22.0	9.0	0.003	0.043	2586.67
***Loxl1***	Lysyl oxidase-like 1	24.0	3.0	5.92e-09	3.57e-06	2012
***Col4a4***	Collagen, type IV alpha 4	24.0	3.0	0.0005	0.014	1991.64
***Svep1***	Sushi, von Willebrand factor type A	22.0	15.0	0.001	0.024	1968.1
***Mmp9***	Matrix metalloproteinase 9	28.0	1.0	0.0005	0.014	1904.63
***Plxdc2***	Plexin domain containing protein 2	26.0	9.0	6.48e-05	0.003	1853.26
***Col12a1***	Collagen, type XII alpha 1	24.0	15.0	0.0001	0.006	1834.77
***P4ha1***	Prolyl 4-hydroxylase alpha 1	24.0	5.5	0.0003	0.009	1823.97
***S100g***	S100 calcium binding protein G	26.0	4.0	2.19e-06	0.0002	1797.8
***Lama1***	Laminin, alpha 1	28.0	7.5	0.001	0.028	1772.29
***Plod1***	Procollagen-lysine	24.0	17.0	1.57e-05	0.001	1676.2
***Ltbp2***	Latent transforming growth factor beta binding protein 2	22.0	19.0	1.08e-05	0.0008	1423.32
***Timp3***	Tissue inhibitor of metalloproteinase 3	24.0	15.0	1.77e-09	1.46e-06	1138.08

Gene Symbols: Genes of highly expressed proteins in mouse fibrotic lung tissue gathered from MatrisomeDB. Period: How often the cycle is repeated. Phase: The timing in the individual tissues. *p*-value: The probability of the dataset not being cyclic. q-value: The minimum rate at which a gene is mistakenly called cyclic. Confidence score: Scaled distribution score of ECM proteins represented by peptide-to-spectrum matches (PSMs). The 38 genes presented in Table 1 had a confidence score of at least 1% of the greatest confidence score presented by MatrisomeDB.

**Table 2:** **Gene Ontology enrichment analysis for rhythmic mouse ECM and ECM-related protein targets analyzed using STRING DB**.

GO-Term/Pathway	Description	Count in Network	Strength	False Discovery Rate
**Biological Process (GO)**				
GO:0007155	Cell adhesion	17	1.13	1.23E-11
GO:0030198	Extracellular matrix organization	11	1.45	6.23E-10
GO:0009887	Animal organ morphogenesis	14	0.91	1.40E-06
GO:0009888	Tissue development	17	0.77	1.40E-06
GO:0009653	Anatomical structure morphogenesis	18	0.68	6.91E-06
**Molecular Function (GO)**				
GO:0005201	Extracellular matrix structural constituent	10	2.02	3.45E-14
GO:0046872	Metal ion binding	22	0.57	3.77E-06
GO:0005178	Integrin binding	6	1.42	8.10E-05
GO:0005509	Calcium ion binding	9	0.95	0.00029
GO:0050840	Extracellular matrix binding	4	1.61	0.0017
**Cellular Component (GO)**				
GO:0062023	Collagen-containing extracellular matrix	31	1.71	4.87E-46
GO:0031012	Extracellular matrix	32	1.61	2.80E-45
GO:0005576	Extracellular region	35	0.97	5.75E-30
GO:0005604	Basement membrane	15	1.93	1.54E-22
GO:0005615	Extracellular space	20	0.92	3.31E-12
**KEGG Pathways**				
mmu04512	ECM-receptor interaction	10	1.84	1.74E-13
mmu04974	Protein digestion and absorption	7	1.59	1.08E-07
mmu05146	Amoebiasis	7	1.6	1.08E-07
mmu04510	Focal adhesion	8	1.39	1.18E-07
mmu05165	Human papillomavirus infection	8	1.14	6.31E-06

## Data Availability

This manuscript is a review article and therefore, data sharing is not applicable.

## Abbreviations

αSMA: alpha smooth muscle actin; CF, cystic fibrosis; CLOCK, circadian locomotor output cycles protein kaput; COPD, chronic obstructive pulmonary disease; CRY, cryptochrome; CS, cigarette smoke; BMAL1, brain and muscle ARNT-like 1; ECM, extracellular matrix; EMT, epithelial-to-mesenchymal transition; EV, extracellular vesicles; FMT, fibroblast-to-myofibroblast transition; GSK3β, glycogen synthase kinase-3 beta; IL-6, interleukin 6; IPF, idiopathic pulmonary fibrosis; KO, knockout; LOX, lysyl oxidase; MMP, matrix metalloproteinase; PAH, pulmonary arterial hypertension; PER, period; PSMs, peptide-to-spectrum matches; REV-ERBα/NR1D1, nuclear receptor subfamily 1group D member 1; ROR, retinoid-related orphan receptor; SCN, suprachiasmatic nucleus; SMAD2/3, SMAD family member 2/3; TGFβ, transforming growth factor beta; TIMP, tissue inhibitor of metalloproteinase; WT, wild-type.
